# Sleep and Sleepiness Measured by Diaries and Actigraphy among Norwegian and Austrian Helicopter Emergency Medical Service (HEMS) Pilots

**DOI:** 10.3390/ijerph19074311

**Published:** 2022-04-04

**Authors:** Tine Almenning Flaa, Bjørn Bjorvatn, Ståle Pallesen, Erik Zakariassen, Anette Harris, Pia Gatterbauer-Trischler, Siri Waage

**Affiliations:** 1 Department of Research and Development, The Norwegian Air Ambulance Foundation, 0184 Oslo, Norway; 2 Department of Global Public Health and Primary Care, University of Bergen, 5009 Bergen, Norway; bjorn.bjorvatn@uib.no (B.B.); erik.zakariassen@uib.no (E.Z.); 3 Norwegian Competence Center for Sleep Disorders, 5021 Bergen, Norway; staale.pallesen@uib.no (S.P.); siri.waage@uib.no (S.W.); 4 Department of Psychosocial Science, University of Bergen, 5015 Bergen, Norway; anette.harris@uib.no; 5 Optentia, The Vaal Triangle Campus of the North-West University, Vanderbijlpark 1900, South Africa; 6 Department of Air Rescue College, Christophorus Flugrettungsverein, 1030 Vienna, Austria; pia.gatterbauer-trischler@oeamtc.at

**Keywords:** sleep, sleepiness, shift work, long working hours, successive shifts, HEMS, air ambulance, somnolence, fatigue

## Abstract

The study examined sleep and sleepiness among shift working Helicopter Emergency Medical Service pilots from Norway (Norwegian Air Ambulance; NAA) and Austria (Christophorus Flugrettungverein; CFV). Both pilot groups (N = 47) worked seven consecutive 24 h shifts. Sleep was assessed by diaries and actigraphy while sleepiness was assessed by the Karolinska Sleepiness Scale, all administered throughout the workweek. The results indicated that all pilots had later bedtime (*p* < 0.05) and wake-up time (*p* < 0.01) as they approached the workweek end, but no change during the workweek was evident regarding wake after sleep onset, time in bed, total sleep time, or sleep efficiency. The NAA pilots had later bedtime (*p* < 0.001) and wake-up time (*p* < 0.001), spent more time awake after sleep onset (*p* < 0.001), more time in bed (*p* < 0.001), slept longer (*p* < 0.01), and had lower sleep efficiency (*p* < 0.001) compared with the CFV pilots. The sleepiness levels of all pilots were slightly elevated on the first workday but lower on the following workdays (^day 2^ *p* < 0.001, ^day 3^ *p* < 0.05). For both pilot groups, no major change in sleep or sleepiness parameters throughout the workweek was detected. The NAA pilots reported somewhat more disturbed sleep but obtained more sleep compared with the CFV pilots.

## 1. Introduction

Shift work is denoted as work that takes place at irregular hours, including evenings, nights, and weekends [1]. Long working hours represent one aspect of shift work and is often defined as work that exceeds 48 h per week [2]. Shift work and long working hours are associated with sleep loss, decreased job performance, and an increased rate of occupational accidents [3,4].

The Helicopter Emergency Medical Service (HEMS) represents a component of aviation whose primary function is to serve patients with severe disease or trauma, by providing rapid transport to hospital and/or transport of an anesthesiologist and HEMS crew members to the site [5,6]. HEMS is an on-demand service with an inherent urgency when missions occur. This entails taking on missions at any hour with potential challenging weather conditions, and operations are often conducted in rough and hostile terrains with an increased risk of accidents [7,8]. In Europe, the organization of HEMS varies in terms of scheduling and setting, ranging from 12 h shifts to seven consecutive 24 h shifts [9,10]. The Norwegian Air Ambulance (NAA) and the Christophorus Flugrettungverein (CFV) in Austria are two of few HEMS operators in Europe that share the same seven consecutive 24 h shift schedule, where the workers are stationed at the base during the workweek. However, the NAA operates 24/7 in contrast to the CFV that only takes on missions during daylight [10]. The latitude in Norway ranges from 58°03′ N to 70°66′ N while the Austrian latitude ranges from 46°53′ N to 48°82′ N.

The limited body of research that exists on sleep in HEMS shows ambiguous findings. As sleep loss and sleepiness are notable occupational hazards in aviation [11], more knowledge about sleep in HEMS is warranted. In terms of shift arrangement, one study suggested that a 7-day work period resulted in cumulative sleep loss of approximately 15 h [12], whereas another study found only 30 min of sleep debt following 24 h shifts [13]. An American study found poor sleep quality in 50% of the workers during 12 h and 24 h shifts. Nevertheless, they found no change in cognitive performance during the same shifts [14]. However, 84% of HEMS pilots working at least three consecutive 12 h day shifts reported that fatigue had affected their flight performance. Half of the pilots reported obtaining 4 h or more of sleep per day [15]. Previous findings for Norwegian HEMS crew revealed low sleepiness levels and a daily average of 7 h of sleep during workweeks. However, wake after sleep onset was longer and sleep efficiency was lower during the workweek, compared with weeks off work [16,17]. A comparative study indicated that there exist some differences between the NAA and CFV pilots regarding use of sleepiness strategies and elements that prevented napping at work. To prevent sleepiness, the NAA pilots napped and exercised, while the CFV pilots generally kept busy. Furthermore, the CFV pilots more frequently reported that environmental factors, phone calls, and administrative duties prevented napping [10].

Consequently, more research of sleep and sleepiness measures in relation to work hours in this occupational group is deemed necessary. A multicenter design provides a unique opportunity to gain insight into how the work setting and schedule affect the workers’ sleep. Thus, the aim of the present study was to examine and compare sleep and sleepiness in HEMS pilots in two European countries working 24 h shifts for seven consecutive days. In addition, we examined whether the number and/or duration of missions affected sleep and sleepiness across the two pilot groups.

## 2. Materials and Methods

### 2.1. Participants

The study was conducted in Norway during the spring/summer of 2015 and in Austria during the spring/summer of 2016. For the present data collection, 29 pilots working on all nine Norwegian bases operated by the Norwegian Air Ambulance were invited to participate. Of these, 25 provided data that were eligible for analyses, yielding a response rate of 86%. A collaboration with the Austrian Christophorus Flugrettungverein was established in order to conduct a multicenter study on HEMS pilots with the same work schedule and using the same instruments. In Austria, 24 CFV pilots were invited to participate, comprising a representative sample of both rural and urban bases. Of these, 22 CFV pilots provided data that were eligible for inclusion, yielding a response rate of 92%.

### 2.2. Procedure

For the NAA pilots, the shift schedule comprised a 7 day work period followed by 14 days off work, then a new 7 day work period followed by 21 days off work. The workweek started and ended at 10:00 h on the first and seventh workday, respectively. The NAA takes on missions at all hours throughout the year. During the workweek, the pilots live together with the crew on a base that is fully equipped with all necessary facilities, including separate bedrooms with private bathrooms. For the CFV pilots, the shift schedule consisted of a 7-day work period followed by 7 days off work, then a new 7-day work period and 7 days off work. The workweek started at sunrise (06:00 h earliest) on the first workday and ended at sunset (21:00 h latest) on the seventh workday. The workers in Austria only perform missions initiated at daylight. During the workweek, the CFV pilots live together with the crew on a base, equipped with single bedrooms and enclosed private bathrooms. Those who live in proximity to the base (<30 min) have the option of spending the night at home. The fatigue risk management systems include flight and duty time limitations approved by the Civil Aviation Authority in Norway for NAA and the Austrian National Aviation Authority (Austro Control) for CFV (for details, see [10]).

### 2.3. Measures

#### 2.3.1. Questionnaire

A questionnaire assessing demographic and background information was completed on the first workday. It included information regarding sex, age, years in position, caffeine intake at work (number of cups), sleep need (h), and the question “do you experience sleep problems related to your work schedule?” (1 = no to 5 = very much).

#### 2.3.2. Mission Log

Both the NAA and CFV staff provided a daily overview of the number of missions and total time spent on missions for each worker. The total time spent on missions was recorded from the time an alarm initiated a mission until the workers landed back at base. As the NAA pilots also performed training sessions during the workweek, time spent on this activity was included in their data in order to calculate the total workload.

#### 2.3.3. Sleep Diary

The sleep diary was completed upon awakening in the morning on all seven workdays. Sleep variables such as bedtime, wake-up time, wake after sleep onset, time in bed, total sleep time, and sleep efficiency (total sleep time/time in bed × 100) were calculated based on the diaries. In total, 45 out of 47 participants completed the sleep diary during the workweek.

#### 2.3.4. Actigraphy

The workers wore an actigraph (Actiwatch 2, Respironics Inc., Cambridge, MA, USA), a device with the size and appearance of a watch, on the non-dominant wrist that records activity and movement continuously [18]. Based on these data, algorithms estimate sleep and wake profiles. The actigraphs recorded these individual profiles in 1 min epochs applying the default (medium) threshold for sleep/wake detection provided by the Actiware software. For the medium threshold, the number of activity counts used to identify wake (sensitivity threshold) was 40 counts per epoch, whereas the sleep start/end threshold was 10 min of inactivity/activity. The workers were instructed to wear the actigraph continuously throughout the workweek. The scoring was based on the steps proposed by Chow and colleagues [19], including a hierarchical approach first emphasizing event markers and activity levels. In the cases where the event marker was absent (35% of NAA observations/18% of CFV observations), sleep diary and activity levels were used to set sleep times. Bedtime, wake-up time, wake after sleep onset, time in bed, total sleep time, and sleep efficiency were calculated by the Actiware software. Due to technical problems, data from one actigraph was excluded from the analysis.

#### 2.3.5. Karolinska Sleepiness Scale

Sleepiness was assessed by the Karolinska Sleepiness Scale (KSS) [20], which is a 9-point scale measuring subjective sleepiness (1 = very alert to 9 = very sleepy, great effort to stay awake, fighting sleep). Scoring seven or more indicates excessive sleepiness. The participants completed the KSS every other hour while awake throughout the workweek. Mean scores were extracted for the individual workday throughout the workweek but scores at every time point (every second hour awake between 08:00–00:00 during the workdays) were also noted. The KSS was completed by all 47 participants during the data collection period.

### 2.4. Data Analyses

Categorical variables are presented as numbers and percentages; *n* (%), whereas continuous variables are presented as mean (standard deviation [SD]) for symmetric data, and median (quartiles) for asymmetric data. The statistical analyses were conducted using Stata 16.1. Regarding the question about sleep problems related to the work schedule, none of the pilots ticked for the response options ‘much’ or ‘very much’. The response alternatives ‘very much’, ‘much’, ‘a little’, and ‘some’ were merged into ‘yes’, and the variable was dichotomized into ‘yes’ and ‘no’ before conducting statistical analysis. Fisher’s exact test was applied to examine differences between the countries in terms of sleep problems and sex, due to tables containing cells with a frequency <5 and a low sample size. Student’s *t*-tests were applied to explore differences in age, sleep need, and caffeine intake, whereas Mann–Whitney U tests were used to compare differences in median values regarding years in position between the countries. The latter was also applied for analysis pertaining to the number and duration of missions. Linear mixed models (LMM) were used to analyze the sleep and sleepiness variables over the course of a workweek, both with NAA and CFV collapsed and separately for each crew. Specifically, the sleep variables bedtime, wake-up time, wake after sleep onset, time in bed, total sleep time, and sleep efficiency based both on sleep diaries and actigraphy were included as dependent variables. The KSS was fitted as the dependent variable for exploring sleepiness. Day (1–7) and time (only KSS; 08:00–00:00 h) were included in the models as a fixed factor with day 1 and 08:00 h as references, whereas subjects were included as a random factor in all analyses. Number of missions and time spent on missions were inserted as covariates. The statistical significance level was set at *p* < 0.05.

### 2.5. Missing Data 

Both sleep diary data and KSS data were marked as missing if the worker failed to fill out certain time points or columns and when technical issues prevented data collection or extraction from the actigraphs. For all variables, the number of missing entries was not higher than 6%.

### 2.6. Ethics

The study protocol was reviewed and approved by the Regional Committee for Medical and Health Research Ethics, health region West, Norway (no. 2014/593). No additional ethical approval was needed by the corresponding Austrian authorities (415-EP/73/671-2016).

## 3. Results

### 3.1. Descriptive Statistics

Descriptive data are presented in Table 1.

Twenty-five NAA pilots (24 males/one female) and 22 CFV pilots (all males) participated in the study. The mean age was 43.6 (*SD* = 5.2) years for the NAA pilots and 42.8 (*SD* = 6.1) years for the CFV pilots. There was no significant age difference between the two crews (*t* = 0.457, *p* = 0.650). Similarly, no differences were evident between the pilot groups regarding sleep need (*p* = 0.225) or sleep problems related to work schedule (*p* = 0.144). However, the NAA pilots reported drinking more caffeinated beverages at work compared with the CFV pilots (*p* < 0.01), while the CFV pilots had more years in the same position compared with the NAA pilots (*p* < 0.05).

### 3.2. Number of Missions and Time Spent on Missions

For the NAA pilots, the median (quartiles) number of missions during the workweek was 19 (13–22), ranging from 7–30, whereas the corresponding numbers for the CFV pilots were 26 (21–30), ranging from 15–51, which amounted to a significant difference in the pilot groups (*z* = −3.25, *p* < 0.01). In total, 42 (9%) of the NAA pilots’ missions were classified as night missions (between 00:00–07:00 h). For the NAA pilots, the median (quartiles) time spent on missions during the workweek was 23.3 h (19.1–32.9), ranging from 13.2–48.0, and 23.7 h (19.4–29.5), ranging from 15.4–58.3, for CFV pilots, however, this difference was not significant (*z* = −0.023, *p* = 0.981).

### 3.3. Sleep Measured throughout the Workweek

**Figure 1:**
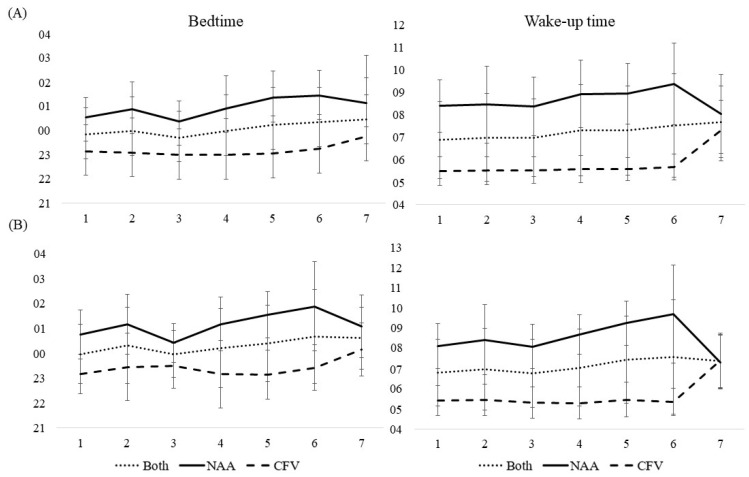
Mean scores of bedtime and wake-up time during a 7-day workweek among pilots in the Norwegian Air Ambulance (NAA; *n* = 25), Christophorus Flugrettungverein (CFV; *n* = 22), and both groups collapsed, measured by (**A**) sleep diary and (**B**) actigraphy. Error bars represent standard deviations.

**Figure 2:**
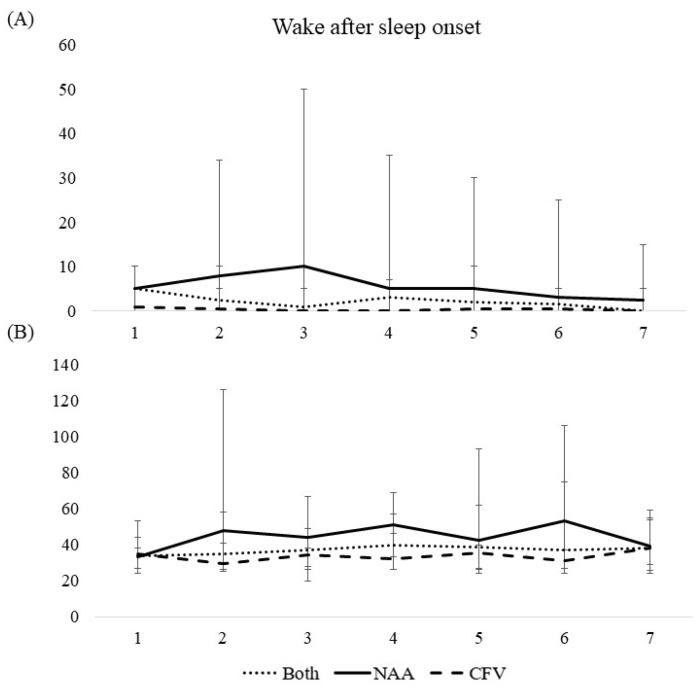
Median scores of wake after sleep onset during a 7-day workweek among pilots in the Norwegian Air Ambulance (NAA; *n* = 25), Christophorus Flugrettungverein (CFV; *n* = 22), and both groups collapsed, measured by (**A**) sleep diary and (**B**) actigraphy. Error bars represent quartiles.

**Figure 3:**
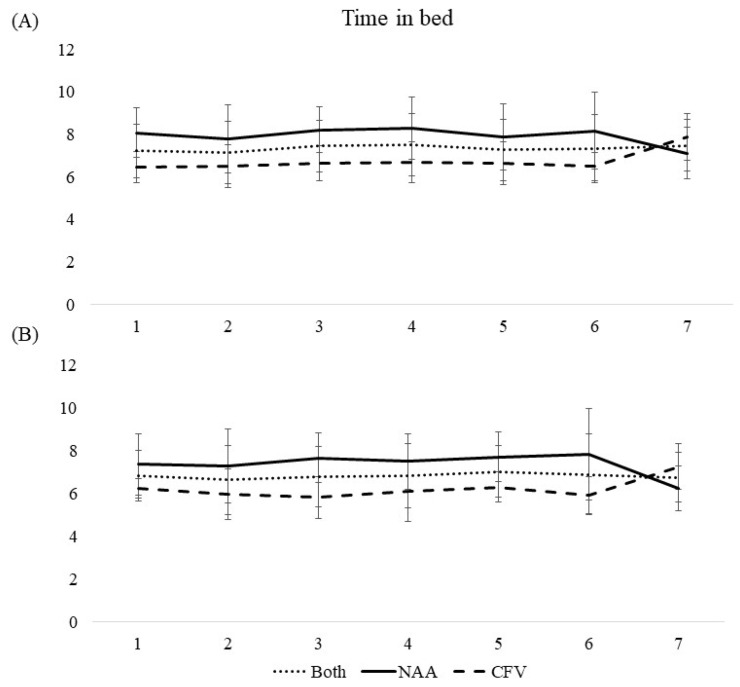
Mean scores of time in bed during a 7-day workweek among pilots in the Norwegian Air Ambulance (NAA; *n* = 25), Christophorus Flugrettungverein (CFV; *n* = 22), and both groups collapsed, measured by (**A**) sleep diary and (**B**) actigraphy. Error bars represent standard deviations.

**Figure 4:**
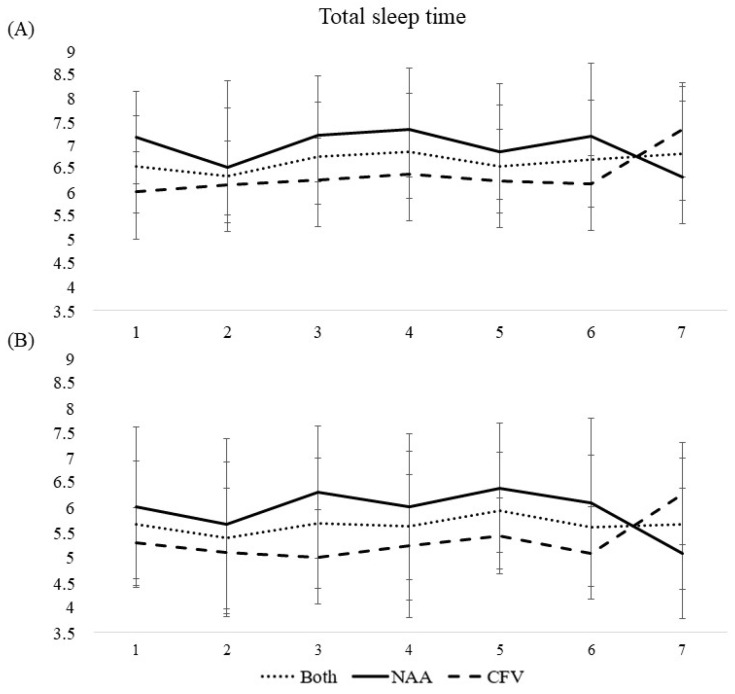
Mean scores of total sleep time during a 7-day workweek among pilots in the Norwegian Air Ambulance (NAA; *n* = 25), Christophorus Flugrettungverein (CFV; *n* = 22), and both groups collapsed, measured by (**A**) sleep diary and (**B**) actigraphy. Error bars represent standard deviation.

**Figure 5:**
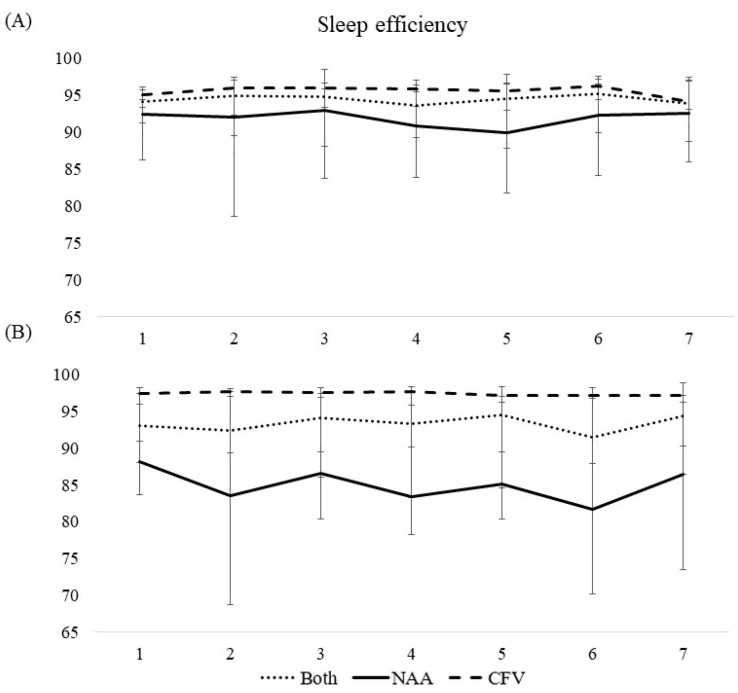
Median scores of sleep efficiency during a 7-day workweek among pilots in the Norwegian Air Ambulance (NAA; *n* = 25), Christophorus Flugrettungverein (CFV; *n* = 22), and both groups collapsed, measured by (**A**) sleep diary and (**B**) actigraphy. Error bars represent quartiles.

Sleep diary and actigraphy data are presented in Table 2 and Table 3 and Figure 1, Figure 2, Figure 3, Figure 4 and Figure 5. 

#### 3.3.1. Bedtime and Wake-Up Time

For both pilot groups collapsed, bedtime and wake-up time measured with sleep diaries were consistent throughout the workweek, except for day 7 which had later bedtime and wake-up time compared with the reference day (day 1) (Table 2, Figure 1). Bedtime and wake-up time measured with actigraphy revealed a consistency the first five days, followed by later bedtime and wake-up time on day 6 and 7 compared with the reference day (Table 2, Figure 1). A difference between the pilot groups was evident, where NAA pilots had later bedtime (^diary NAA^00:57 h vs. ^CFV^23:10 h, *b* = −1.67, *p* < 0.001, ^actigraphy NAA^01:09 h vs. ^CFV^23:26 h, *b* = −1.73, *p* < 0.001) and wake-up time (^diary NAA^08:38 h vs. ^CFV^05:47 h, *b* = −2.73, *p* < 0.001, ^actigraphy NAA^08:32 h vs. ^CFV^05:40 h, *b* = −2.85, *p* < 0.001) compared with the CFV pilots.

For the NAA pilots, there was no change in bedtime or wake-up time throughout the workweek as measured with the sleep diary. Bedtime and wake-up time measured with actigraphy revealed a consistency the first four days of the workweek, but were later on day 5 and 6, whereas wake-up time was earlier on day 7 compared with the reference day (Table 2, Figure 1). The number of missions neither affected bedtime or wake-up time, but the duration of missions was positively associated with later bedtime (assessed with both sleep diary and actigraphy) and wake-up time (sleep diary) (Table 3). For the CFV pilots, no change in bedtime and wake-up time throughout the workweek was evident, except for day 7 which was delayed compared with the reference day (Table 2, Figure 1). The number of missions did not affect bedtime or wake-up time. The duration of missions was positively associated with later bedtime (sleep diary and actigraphy) and wake-up time (actigraphy) (Table 3).

#### 3.3.2. Wake after Sleep Onset, Time in Bed, Total Sleep Time, and Sleep Efficiency

Wake after sleep onset, time in bed, total sleep time, and sleep efficiency were all consistent throughout the workweek for both pilot groups collapsed (Table 2, Figure 2, Figure 3, Figure 4 and Figure 5). A difference between the pilot groups was evident, where the NAA pilots spent more time awake after sleep onset (^diary NAA^00:05 h vs. ^CFV^00:00 h, *b* = -.417, *p* < 0.001, ^actigraphy NAA^00:43 h vs. ^CFV^00:34 h, *b* = −0.498, *p* < 0.001), more time in bed (^diary NAA^07:56 h vs. ^CFV^06:45 h, *b* = −1.17, *p* < 0.001, ^actigraphy NAA^07:23 h vs. ^CFV^06:14 h, *b* = −1.12, *p* < 0.001), slept longer (^diary NAA^06:58 h vs. ^CFV^06:22 h, *b* = −0.508, *p* < 0.01, ^actigraphy NAA^05:58 h vs. ^CFV^05:22 h, *b* = −0.670, *p* < 0.01), and had lower sleep efficiency (^diary NAA^91.7% vs. ^CFV^95.6%, *b* = 7.29, *p* < 0.001, ^actigraphy NAA^85.4% vs. ^CFV^97.3%, *b* = 17.0, *p* < 0.001) compared with the CFV pilots. For the NAA pilots, no change in wake after sleep onset or sleep efficiency was evident throughout the workweek. Time in bed and total sleep time were consistent throughout the workweek, except for day 7 where the scores were lower compared with the reference day (Table 2). The number of missions neither affected wake after sleep onset or sleep efficiency, however, the duration of missions was negatively associated with wake after sleep onset (sleep diary) and positively associated with sleep efficiency (sleep diary). Neither number of missions or duration of missions affected time in bed and total sleep time (Table 3).

For the CFV pilots, no changes in wake after sleep onset or sleep efficiency were evident throughout the workweek. Time in bed and total sleep time were consistent throughout the workweek, except for day 7 where the scores were higher compared with the reference day (Table 2, Figure 2, Figure 3, Figure 4 and Figure 5). Number of missions was positively associated with sleep efficiency (actigraphy), but not related to wake after sleep onset, time in bed, or total sleep time. Duration of missions neither affected wake after sleep onset, time in bed, total sleep time or sleep efficiency irrespective of instrument used (Table 3).

### 3.4. Sleepiness Measured throughout the Workweek 

#### Karolinska Sleepiness Scale (KSS)

**Figure 6:**
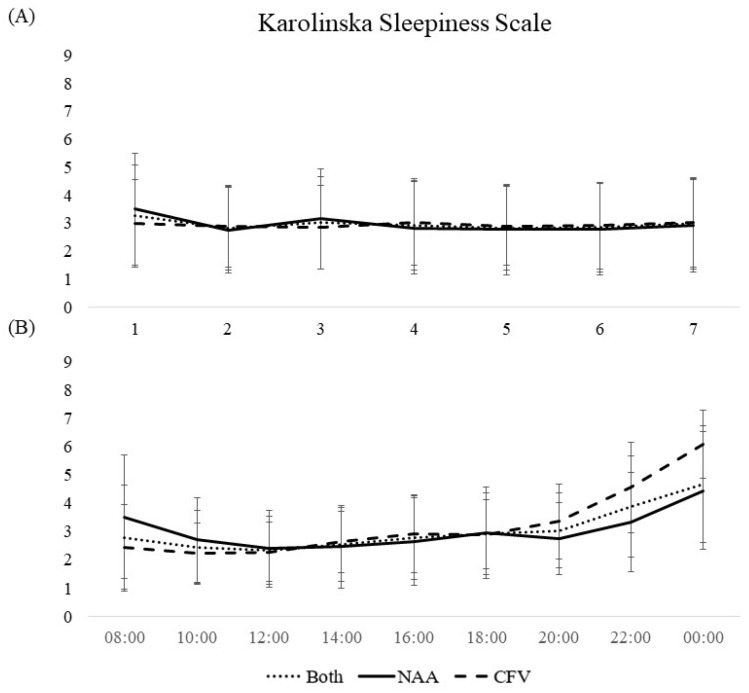
Mean scores of Karolinska Sleepiness Scale among pilots in the Norwegian Air Ambulance (NAA; *n* = 25), Christophorus Flugrettungverein (CFV; *n* = 22), and both groups collapsed. (**A**) During a 7-day workweek and (**B**) between 08:00–00:00 on workdays. Error bars represent standard deviation.

Results regarding sleepiness are presented in Table 4 and Figure 6.

For both pilot groups collapsed, day 2 (*p* < 0.001) and day 3 (*p* < 0.05) had lower KSS scores compared with the reference day (day 1) during the workweek. For time of day, the KSS scores at 10:00 (*p* < 0.05) and 12:00 (*p* < 0.01) were lower, while the scores at 18:00 (*p* < 0.05), 20:00 (*p* < 0.01), 22:00 (*p* < 0.001), and 00:00 (*p* < 0.001) were higher compared with the reference time (08:00 h). No difference between the pilot groups was evident (*b* = −0.039, *p* = 0.814).

## 4. Discussion

The results for both pilot groups collapsed indicated a slight delay in the sleep period towards the end of the workweek. No change throughout the workweek was evident for wake after sleep onset, time in bed, total sleep time, or sleep efficiency. However, several differences between the HEMS contingent on country were present. The NAA pilots had nearly two hours later bedtime and nearly three hours later wake-up time, they spent over an hour more in bed, slept about 40 min more, and had lower sleep efficiency compared with the CFV pilots (on sleep diaries). The sleepiness scores for both pilot groups collapsed were overall low throughout the workweek but were slightly elevated on the first workday compared with the rest of the workweek. Not surprisingly, over the course of a day, the sleepiness levels comprised a slight U-curve including a steady increase towards the end of the day.

The results indicated that bedtime and wake-up times were consistent in the beginning of the workweek and then somewhat delayed towards the last days of the workweek. This delay could be explained by protection from domestic obligations that relieve the pilots of duties involving early wake-up times (i.e., child rearing and/or transportation to work/kindergarten/school). However, one should keep in mind that those CFV pilots who lived less than 30 min from the base could spend their nights at home during the workweek. Furthermore, missions occurring at late hours may delay bedtime, and especially, combined with the absence of early morning missions could explain these findings. However, we do not have detailed information about when the missions occurred, thus more studies are needed to clarify this.

The NAA pilots had clearly later bedtime and wake-up times compared with the CFV pilots. While the CFV pilots only initiated missions during daylight, the NAA pilots also took on missions after dark, which could explain why they went to bed and woke up later than the CFV pilots as night work is known to delay the circadian rhythm [21]. Latitude and daylight exposure could also influence the findings. For example, the average hours of daylight in May in Oslo is 17:14 h compared with 15:16 h in Vienna [22,23]. The difference between the NAA and CFV pilots’ latitude location could therefore at least partly explain these aforementioned effects. The higher number of missions for the CFV pilots could explain earlier wake-up times compared with the NAA pilots. Subsequently, CFV pilots who spent the night at home would have to get up even earlier to reach base before dawn which could contribute further to this discrepancy. The bedtime and wake-up time were positively associated with the duration of missions for both groups. This could indicate that the more time the pilots spent on missions, the more delayed their sleep period would be. This notion is in accordance with a previous HEMS study in Norway, where higher amounts of total work time were associated with later bedtime during the summer season [16].

Time in bed and total sleep time obtained by both pilot groups collapsed were consistent throughout the workweek. Furthermore, the NAA pilots spent more time in bed and had higher total sleep time (close to 7 h per night) compared with the CFV pilots (about 6.5 h per night). As all NAA pilots live on the base during their workweek, this could enable them to spend more time in bed and thus obtain more total sleep time despite also spending more time awake after sleep onset than the CFV pilots. However, there are some uncertainties related to what extent the CFV pilots spend their nights at home as this was not registered.

The self-reported sleep need was 07:01 h for the NAA pilots and 07:16 h for the CFV pilots. The results concerning total sleep time measured with sleep diaries indicated that the NAA pilots obtained approximately the self-reported need, while the CFV pilots obtained less. Total sleep time derived from actigraphy was clearly lower than that measured by the sleep diary, which is a common finding in studies using both instruments [24,25]. Still, the diary findings indicated that most of the pilots obtained the recommended or, at least, appropriate amounts of sleep according to Hirshkowitz and colleagues [26]. This is contrary to other studies that have reported restricted sleep duration when working irregular shifts and long hours [25,27]. However, the discrepancy between the two sleep measures necessitates a cautious interpretation of the results and should be considered in the development of the internal fatigue risk management systems.

The wake after sleep onset and sleep efficiency were consistent throughout the week for both pilot groups collapsed. Furthermore, the NAA pilots spent more time awake after sleep onset and had lower sleep efficiency compared with the CFV pilots. As previously mentioned, the NAA pilots conduct missions after dark, while the CFV pilots do not. Although only 9% of their missions were classified as night missions, this could still explain the greater amount of time spent awake after sleep onset and thus lower sleep efficiency. Additionally, the NAA pilots could receive calls and mission assessments, not resulting in missions, but that required the pilots to wake up. However, detailed data on calls and mission assessments were not available, hence the aforementioned notions are somewhat speculative. Nevertheless, on-call studies have found longer wake time after sleep onset and lower sleep efficiency during on-call nights, even when calls were absent [28,29,30]. This suggests that the mere anticipation of a call or mission could lead to more wakefulness. Hence, both actual and anticipated missions during the sleep period could result in longer wake time after sleep onset and lower sleep efficiency for the NAA pilots.

Interestingly, results for the two pilot groups revealed a convergence on the last workday, where the NAA pilots woke up earlier, spent less time in bed, and had lower total sleep time, whereas the CFV pilots woke up later, spent more time in bed, and had higher total sleep time compared with the previous workdays. The changes in the NAA pilots’ sleep variables could be due to tasks and duties that had to be completed upon the end of the workweek and may also reflect that they lived farther from the base than the CFV pilots and hence had more strenuous travel arrangements ahead. However, one might also expect similar tasks and duties for the CFV pilots, but the opposite finding complicates the interpretation.

The overall sleepiness levels were low throughout the workweek for both pilot groups. As both pilot groups primarily live on the base during the workweek, the base facilities including separate bedrooms could promote good rest in between missions, resulting in low sleepiness levels. This is contrary to previous findings on sleepiness and shift work [31,32]. A study on pilots found, for example, faster accumulation of fatigue in multi-segmented workdays compared with single segmented [33]. Although the CFV pilots conducted more missions than the NAA pilots, there was no difference between the countries regarding sleepiness scores. This may suggest that living on base during the workweek protected against domestic responsibilities that otherwise could cause an increase in sleepiness. This notion was supported by a study that found that 26% of HEMS pilots reported sleep disturbance due to child care [15]. The sleepiness levels were slightly increased on the first workday but remained low on the remaining workdays for all pilots. This could be due to the commute on the first workday, as the workweek started at 10:00 h and at sunrise (earliest 06:00 h) for the NAA and CFV pilots, respectively. Considerable commute time would imply an early morning for the pilots in order to reach the base for shift start. Over the course of a workday, the sleepiness levels were low at the start with an increase towards the end. Rather than being a consequence of the work schedule and setting, this variation in sleepiness resembled the oscillations of normal circadian rhythms [34]. The results on sleepiness corroborate a previous Norwegian HEMS study where both pilots (the same pilots as in the present study) and HEMS crew members reported low sleepiness levels throughout the workweek and over the course of a workday. Interestingly, this previous study also found the sleepiness levels during the workweek to be lower than the weeks off (both preceding and following) work [17]. A recent review found that risk associated with fatigue increases as the workdays exceeds 16 h and coincides with habitual sleep hours [35]. The current sleepiness results indicated that neither the NAA nor the CFV pilots experienced substantial sleepiness throughout the workweek or workdays although the work setting included more than 16 h workdays and work in conflict with habitual sleep hours.

Some strengths and limitations of the present study should be noted. The multicenter and longitudinal design with numerous daily measurements over seven consecutive days provided detailed insight into the oscillation of sleep and sleepiness during the workweek in two different countries with similar shift schedules. This intensive data collection diminished the chance of recollection bias and improve data quality. The study included both subjective and objective measurements which also represent an asset, as it enables thorough documentation of the pilots’ sleep. The objective sleep measures prevent data from being influenced by response biases associated with subjective measures.

The “healthy worker effect” could have influenced our results, as the pilots represent a highly selected and resilient cohort that cope well with the shift schedule and work setting. For this reason, generalizing to other work populations must be conducted with prudence. However, as this occupation constitutes a high-risk operation for pilots, HEMS crew members, and patients, information about how this particular groups’ sleep is affected by the work schedule is of high value. Some discrepancies between sleep diary and actigraphy data were noted and are in line with previous studies [24,36] reflecting different (self-report and movement) data sources. Furthermore, as the HEMS is a male-dominant occupation, the sample expectedly consisted predominantly of males. Thus, generalization of the findings to other populations with a higher proportion of females must be made with caution. The mission data could benefit from a higher level of detail (timing, type, etc.) that would enable a more comprehensive analyses of their impact on sleep and sleepiness. Future studies should include larger sample sizes (i.e., several pilot groups) and several workweeks per pilot in order to generate mission data quantities that enable more sophisticated analyses. Furthermore, future studies would benefit from including objective measures of sleepiness, such as the Multiple Sleep Latency Test (MSLT) [37]. However, the MSLT may be somewhat challenging to conduct in field studies such as the present. Other tests of sleepiness, such as the alpha attenuation test [38], may be more suitable in the field.

Although no major sleep problems were detected among the pilots in the present study, the importance of maintaining good sleep for workers in this occupational group should be emphasized. By utilizing sleep opportunities and maintaining a consistent bed- and rise time that aligns with the circadian rhythm, when possible, the workers may be better prepared to meet the unpredictable workload and workhours comprised by the work setting. Furthermore, fatigue risk management systems should be under continuous development to ensure that the potential negative effects of the shift schedule are attenuated. Specific alertness management programs [39] could be one approach to increase awareness and improve alertness strategies among pilots.

## 5. Conclusions

No substantial changes in sleep or sleepiness parameters throughout the workweek were evident. Differences between the two pilot groups were detected, where the NAA pilots reported more fragmented sleep than the CFV pilots. These differences could be due to missions conducted after daylight by the NAA pilots. Still, both pilot groups reported on average more than 6 h of subjective sleep throughout the workweek which lasted 24 h over 7 consecutive days. This could indicate that the pilots cope well with the shift schedule despite its known risk factors (i.e., night work). Fatigue risk management strategies should nevertheless continue to evolve and tailor mitigating measures to avoid the potential detrimental effects.

## Figures and Tables

**Table 1:** Descriptive statistics of years in position, caffeine intake at work, sleep need, and sleep problems related to work schedule for pilots in The Norwegian Air Ambulance (NAA; *n* = 25) and Christophorus Flugrettungverein (CFV; *n* = 22).

	NAA	CFV	NAA	CFV	NAA	CFV		
	*n* (%)		Mean (SD)		Median (IQR)		Statistic	*p*-Value
Years in position (*n* = 47)					5 (3.5–7)	10.5 (5–15)	−2.18 ^a^	0.029 ^b^
Caffeine intake at work (*n* = 45)			4.58 (0.42)	3.05 (0.36)			2.73 ^c^	0.009 ^d^
0 cups	1 (4.0)	3 (13.6)						
1–2 cups	2 (8.0)	3 (13.6)						
3–4 cups	7 (28.0)	11 (50.0)						
5–6 cups	10 (40.0)	4 (18.2)						
>7 cups	5 (20.0)	1 (4.6)						
Sleep need ^§^ (*n* = 44)			07:01 (00:40)	07:16 (00:38)			−1.23 ^c^	0.225 ^d^
Sleep problems related to work schedule (*n* = 45)								0.231 ^e^
Yes	7 (28.0)	11 (50.0)						
No	18 (72.0)	11 (50.0)						

^§^ hh:mm, ^a^ z value, ^b^ *p*-value based on Mann–Whitney U, ^c^ *t*-value, and ^d^ *p*-value based on Student’s *t*-test, ^e^ *p*-value based on Fisher’s exact test.

**Table 2:** Unstandardized beta coefficients for sleep variables in pilots from the Norwegian Air Ambulance (NAA; *n* = 25) and Christophorus Flugrettungverein (CFV; *n* = 22) during a workweek, derived from sleep diary and actigraphy.

Sleep Diary																		
	Bedtime			Wake-Up Time			Wake after Sleep Onset			Time in Bed			Total Sleep Time			Sleep Efficiency		
	Both	NAA	CFV	Both	NAA	CFV	Both	NAA	CFV	Both	NAA	CFV	Both	NAA	CFV	Both	NAA	CFV
Day																		
1 ^§^																		
2	0.043	0.115	−0.056	−0.021	−0.092	−0.008	0.170	0.365	0.007	−0.086	−0.238	0.054	−0.255	−0.707	0.126	−2.18	−6.50	1.31
3	−0.267	−0.412	−0.149	−0.002	−0.077	−0.021	0.065	0.177	0.001	0.273	0.322	0.187	0.215	0.099	0.201	−0.471	−2.65	0.383
4	−0.036	0.087	−0.188	0.218	0.352	0.049	0.049	0.134	−0.021	0.288	0.317	0.255	0.257	0.122	0.369	0.109	−2.11	1.93
5	0.196	0.467	−0.118	0.223	0.328	0.025	0.024	0.084	0.010	0.049	−0.101	0.174	−0.032	−0.364	0.196	−1.06	−3.42	0.126
6	0.347	0.464	0.142	0.451	0.673	0.168	0.099	0.238	−0.006	0.113	0.253	0.027	0.084	0.032	0.154	0.261	−2.20	2.06
7	0.524 *	0.364	0.638 *	0.644 **	−0.519	1.80 ***	−0.094	−0.154	−0.001	0.232	−0.875 *	1.40 ***	0.213	−0.858 *	1.31 ***	−0.900	−2.55	0.115
**Actigraphy**																		
	Bedtime			Wake-up time			Wake after sleep onset			Time in bed			Total sleep time			Sleep efficiency		
	Both	NAA	CFV	Both	NAA	CFV	Both	NAA	CFV	Both	NAA	CFV	Both	NAA	CFV	Both	NAA	CFV
Day																		
1 ^§^																		
2	0.287	0.304	0.288	0.135	0.246	0.036	0.211	0.361	0.075	−0.148	−0.032	−0.255	−0.249	−0.352	−0.183	−1.78	−3.94	0.264
3	−0.089	−0.429	0.311	−0.110	−0.092	−0.098	0.032	0.080	0.024	−0.010	0.363	−0.409	0.038	0.276	−0.319	0.485	0.100	0.331
4	0.084	0.215	−0.050	0.148	0.435	−0.145	0.132	0.210	0.034	0.069	0.244	−0.097	0.007	0.073	−0.034	−0.888	−1.84	0.246
5	0.257	0.583 *	−0.052	0.522	1.01 **	0.022	0.086	0.141	0.057	0.274	0.457	0.073	0.322	0.443	0.110	0.802	0.954	0.193
6	0.564 **	0.801 **	0.307	0.685 *	1.36 **	−0.001	0.219	0.397	−0.014	0.132	0.627	−0.314	−0.012	0.281	−0.182	−1.30	−2.38	0.467
7	0.555 **	0.098	1.02 ***	0.539 *	−0.960 *	2.05 ***	−0.020	−0.197	0.142 *	−0.011	−1.02 *	1.03 ***	0.051	−0.861 *	1.00 **	−0.428	−0.717	0.084

* *p* < 0.05, ** *p* < 0.01, and *** *p* < 0.001. ^§^ Represents the reference groups. The ‘both’ category refers to both pilot groups collapsed.

**Table 3:** The effect of missions on sleep variables in pilots from the Norwegian Air Ambulance (NAA; *n* = 25) and Christophorus Flugrettungverein (CFV; *n* = 22), derived from sleep diary and actigraphy, reported in unstandardized beta coefficients.

	Sleep Diary											
	Bedtime		Wake-Up Time		Wake after Sleep Onset		Time in Bed		Total Sleep Time		Sleep Efficiency	
	NAA	CFV	NAA	CFV	NAA	CFV	NAA	CFV	NAA	CFV	NAA	CFV
Number ^§^	−0.061	−0.052	−0.126	0.006	0.073	0.002	−0.048	0.023	−0.158	0.062	−1.26	0.540
Duration ^∫^	0.003 ***	0.002 *	0.003 **	0.001	−0.001 *	−0.000	−0.000	−0.001	0.002	−0.001	0.023 **	−0.005
	**Actigraphy**											
	Bedtime		Wake-up time		Wake after sleep onset		Time in bed		Total sleep time		Sleep efficiency	
	NAA	CFV	NAA	CFV	NAA	CFV	NAA	CFV	NAA	CFV	NAA	CFV
Number ^§^	0.017	−0.050	−0.010	−0.032	0.093	−0.019	−0.020	0.017	−0.154	0.083	−1.03	0.187 *
Duration ^∫^	0.002 **	0.002 **	0.002	0.002 *	−0.001	−0.000	−0.001	−0.001	0.001	−0.001	0.008	−0.001

* *p* < 0.05, ** *p* < 0.01, and *** *p* < 0.001. ^§^ Number of missions. ^∫^ Duration of missions.

**Table 4:** Unstandardized beta coefficients for Karolinska Sleepiness Scale scores throughout the workweek (7 days) and during workdays (08:00–00:00) in pilots from the Norwegian Air Ambulance (NAA; *n* = 25) and Christophorus Flugrettungverein (CFV; *n* = 22).

	Both	NAA	CFV
Day			
1 ^§^			
2	−0.452 ***	−0.732 ***	−0.126
3	−0.313 *	−0.435	−0.177
4	−0.374	−0.683 *	0.018
5	−0.459	−0.757	−0.103
6	−0.474	−0.690	−0.093
7	−0.439	−0.634	−0.143
Time			
08 ^§^			
10	−0.274 *	−0.662 ***	−0.213
12	−0.369 **	−0.943***	−0.159
14	−0.166	−0.885 ***	0.195
16	0.094	−0.656 ***	0.485 ***
18	0.221 *	−0.399 *	0.469 ***
20	0.350 **	−0.581 **	0.943 ***
22	1.18 ***	0.006	2.11 ***
00	2.08 ***	1.16 ***	3.35 ***

* *p* < 0.05, ** *p* < 0.01, and *** *p* < 0.001. ^§^ Represents the reference groups. The ‘both’ category refers to both pilot groups collapsed.

## Data Availability

The data are available upon reasonable request.
